# *Puerariae lobatae* Radix aqueous extract ameliorates asymptomatic hyperuricemia in a potassium oxonate-induced rat model by dual modulation of uric acid production and excretion

**DOI:** 10.3389/fnut.2025.1685674

**Published:** 2025-11-18

**Authors:** Danping Zhao, Yuannan Wang, Tingting He, Yingna Chen, Yu Bai, Yuchun Huang, Kaiyue Ding, Junnan Ma, Lin Zhang

**Affiliations:** 1 Institute of Integrative Medicine, Dalian Medical University, Dalian, China; 2 The Second Hospital of Dalian Medical University, Dalian, China; 3 The First Affiliated Hospital of Dalian Medical University, Dalian, China; 4 Dalian Institute of Chemical Physics, The Chinese Academy of Sciences, Dalian, China

**Keywords:** *Puerariae lobatae* Radix, hyperuricemia, intestinal flora, network pharmacology, negative feedback regulatory

## Abstract

**Background:**

*Puerariae lobatae* Radix (PLR) is a well-known traditional Chinese medicine and edible natural nutrient, with diverse biological activities, including anti-diabetes, anti-inflammatory, anti-oxidant and liver protection. However, the effects and underlying mechanisms of PLR in hyperuricemia (HUA) are unclear.

**Methods:**

The present study focused on the regulatory effects of aqueous extract from PLR on the asymptomatic hyperuricemia rat model, induced by potassium oxonate. Serum uric acid (SUA), serum blood urea nitrogen (BUN), creatinine (CRE), serum inflammatory factors, anthine oxidase activity, hepatic and renal tissue morphology were measured to assess the anti-hyperuricemia effect. After which, 16SrDNA sequencing and the UHPLC-Q-Orbitrap-MS/MS with network pharmacology, qRT-PCR and molecular docking were employed to elucidated the potential mechanism.

**Results:**

PLR treatment led to a significant improvement in HUA rats, including lower SUA, BUN, CRE and serum inflammatory factors (TNF-*α*, IL-6, IL-1*β*, and NF-κB); inhibited xanthine oxidase activity like xanthine oxidase (XOD), Adenosine deaminase (ADA), regulated the abundance of Firmicutes, Actinobacteriota and Bacteroidota. And the network pharmacological analysis combined with qRT-PCR and molecular docking revealed 4 active compounds of PLR, including hispidulin, cirsimaritin, galangin, and diosmetin, that act on HUA therapeutic targets, like CASP3, NF-κB, PTGS2, PARP1 and JAK2.

**Conclusion:**

Our finding suggest that PLR could effectively ameliorate HUA symptoms by modulating multiple compounds, targets, and pathways. Specifically, hispidulin, cirsimaritin, galangin, and diosmetin are proposed as the key active ingredients in PLR for HUA alleviation. The primary mechanism involves inhibiting xanthine oxidase activity to reduce UA production, promoting UA excretion by restoring the abundance of intestinal flora, and eliminating the negative feedback regulatory mechanism of renal tissue. This study provided a new perspective for the precise exploitation of PLR as a functional food.

## 1 Introduction

Hyperuricemia (HUA) is formed by the disorder of purine metabolism, and marked by overproduction or insufficient excretion of uric acid (UA) (1). As a metabolic disease, the formation of HUA is closely related to the alterations in diet structure and lifestyle, like excessive intake of purine-rich foods, sugar-sweetened drinks, obesity and alcohol consumption (2). As report shown the prevalence of HUA is 2.6–36%, with an upward trend in global, and which is higher in the developed countries especially in coastal areas (3). In China, the prevalence is 6.4% among middle-aged and older Chinese (4), and 17.4% among the general population of the Chinese mainland (5), there is a notable trend of younger individuals. This demographic shift is anticipated to impose a substantial strain on China’s healthcare infrastructure in the foreseeable future. Initial stage of HUA characterized by elevated serum uric acid levels, and without the specific clinical symptoms, also called asymptomatic hyperuricemia (AH) (6). AH is a non-pathological condition, which is defined as serum uric acid level > 6.2 mg/dL, 7 mg/dL in female and male (7). Persistently elevated serum levels in AH lead to the formation of monosodium urate crystalsx, causes chronic arthritis, known as gout (8). In addition, a substantial reports indicate that HUA is a significant risk factor for diabetes hypertension, hyperlipidemia, cardiovascular disease, kidney disease and so on (9, 10). Currently, the clinical approach to managing HUA centers on three aspects. Firstly, the inhibition of UA synthesis via XOD inhibitors. Secondly, the facilitation of uric acid excretion. Lastly, the enhancement of uric acid metabolic decomposition. Prominent pharmacological agents embodying these strategies encompass allopurinol, febuxostat, benzbromarone, rasburicase among others (11). Despite the potential therapeutic advantages, the clinical utility of medications is often constrained by the occurrence of adverse reactions, including allergic reactions, gastrointestinal discomfort, hepatic and renal toxicities as well as cardiovascular complications (12, 13). Consequently, there is an urgent need to explore natural herbal alternatives that may offer therapeutic efficacy for the treatment of HUA while minimizing the risk of adverse effects.

The medical and edible homologous traditional Chinese medicine (TCM) has garnered significant attention in treating HUA, attributed to its favorable safety and demonstrated therapeutic efficacy (14, 15). *Puerariae lobatae* Radix (PLR), derived from the dried root of the leguminous plant *Pueraria lobata* (Willd.) Ohwi, is a traditional medicinal and edible plant that has been utilized in China (Ge-Gen in Chinese), Southeast Asia, and Australia for millennia (16, 17). In China and Japan, PLR has long been revered as a vital edible natural resource, colloquially referred to as “Asian ginseng,” “longevity powder,” and “Royal Special Food.” As documented in the Chinese Pharmacopeia, PLR possesses properties that clearing heat, generating fluids, inducing diuresis, dredging meridians, and alleviating stiffness, and has been used as an antipyretic, diaphoretic, wasting-thirst agent (18). In recent years, PLR has been increasingly incorporated into various health foods, such as soft sweets, beverages, tea, bread (19, 20), due to its rich nutrient composition, including flavonoids, polysaccharides, saponins, triterpenoids, alkaloids and amino acids. These nutrients contribute to its excellent performance in regulating blood circulation, reducing alcohol dependence, hypotensive, hypoglycemic, hypolipidemic effect (21, 22). Recent studies have demonstrated that puerarin and pueraria isoflavones exhibit potent antihyperuricemic activity through the inhibition of XOD, urate transporter and inflammatory (23, 24). Furthermore, PLR is a key component in several Chinese herbal formulas which have uric acid-lowering effect, such as Gegen Qinlian Decoction, Fuling-Zexie Formula (25, 26). These documented bioactivities align with the key pathological mechanisms of hyperuricemia and lending further credence to PLR’s potential anti-hyperuricemia efficacy. Nevertheless, a systematic pharmacological evaluation of PLR specifically for hyperuricemia remains relatively limited, and its underlying mechanisms require further elucidation.

In recent years, with the advancement of microbiomic technologies such as 16S rDNA and metagenomic sequencing, an increasing body of evidence has demonstrated that gut microbiota play a crucial role in the pathogenesis of HUA (27, 28). The elimination of UA in humans is promoted by uricase is facilitated by uricase, however, uricase has been lost as a functional gene during human evolution and is now a “pseudogene.” This congenital genetic deficiency renders individuals highly susceptible to the development of hyperuricemia, influenced by acquired factors such as diet. A recent study has revealed that anaerobic bacteria in the gut microbiota are capable of metabolizing uric acid, maintaining low serum uric acid levels and reducing the risk of gout (29). This finding highlights the compensatory role of the gut microbiota in mitigating the deficiency of the uricase gene and underscores the potential of microbiota-targeted therapies in the treatment of hyperuricemia.

In this research, we aimed to explore the beneficial effects of PLR aqueous extract on HUA rat, and the possible mechanisms were tried to investigate by the integrated strategy of network pharmacology and intestinal flora sequencing. The findings of our research may provide crucial scientific evidence for the preventive and therapeutic potential of PLR in the clinical management of HUA. Additionally, this work offers a novel theoretical foundation for the development of multi-target hypouricaemic agents derived from traditional Chinese medicines.

## 2 Materials and methods

### 2.1 Chemicals and reagents

Potassium oxonate and sodium carboxymethyl cellulose were purchased from Shanghai yuanye Bio-Technology Co., Ltd. (Shanghai, China). Allopurinol was purchased from Shanghai sine wanxiang pharmaceuticals Co., Ltd. (Shanghai, China). The uric acid assay kit was purchased from Biosino Bio-Technology And Science Incorporation (Beijing, China). The blood urea nitrogen (BUN), creatinine (CRE), and Adenosine deaminase (ADA) were purchased from Nanjing Jiancheng Bioengineering Institute (Nanjing, China). The xanthine oxidase (XOD) assay kit was purchased from Elabscience Biotechnology Co., Ltd. The aspartate aminotransferase (AST), alanine aminotransferase (ALT), TNF-*α*, NF-κB, IL-6, IL-1*β* assay kit were purchased from Shanghai Enzyme-linked Biotechnology Co., Ltd. (Shanghai, China).

### 2.2 Preparation of PLR aqueous extract

*Puerariae lobatae* Radix (PLR) was purchased from Bozhou city Jing Wan Chinese medicine slices factory (Bozhou, Anhui, China, Lot 240,301), and was identified by professor Zhang Lin of the institute (College) of Integrative Medicine, Dalian Medical University. PLR occurs as irregular thick slices, thick shreds, or square blocks with a side length of 0.5–1.2 cm. The cut surface is light yellowish-brown to brownish-yellow with a tough texture and prominent fibrous characteristics. It has a slight odor and a slightly sweet taste. The aqueous extract of PLR was prepared by decoction (30). Specifically, PLR was boiled in deionized water at a ratio of 1:12 (w/v) for 1.5 h, followed by two subsequent decoctions in deionized water at a ratio of 1:10 (w/v) for 1 h each. After extraction, the mixture was filtered, concentrated to a final concentration of 1 g crude drug per mL, and store at −80 °C.

### 2.3 Analysis of the constituents in PLR aqueous extract

The components analysis of PLR was conducted using an UPLC-ESI-MS/MS system (UPLC, ExionLC™ AD, https://sciex.com.cn/) and Tandem mass spectrometry system^1^ in positive and negative modes. Briefly, 200 μL of the PLR aqueous extract was mixed with 200 μL of 70% methanol with internal standard extraction solution. The mixture was vortexed for 15 min, followed by centrifugation at 12000 r/min for 3 min at 4 °C. The supernatant was then collected, filtered through a microporous membrane (0.22 μm pore size) and transferred to an injection vial for LC–MS/MS detection. An Agilent SB-C18 column (100 × 2.1 mm, 1.8 μm) was used, with the mobile phase consisting of 0.1% formic acid in pure water (solvent A) and 0.1% formic acid in acetonitrile (solvent B). The column oven temperature was set to 40 °C, and the injection volume was 2 μL. The parameters for the ESI source were as follows: source temperature, 500 °C; ion spray voltage, ±5,500 V (positive/negative ion mode); gas I (GSI), 50 psi; gas II (GSII), 60 psi; curtain gas (CUR), 25 psi; and collision-activated dissociation (CAD), high.

### 2.4 HUA rat model and intervention experiments

#### 2.4.1 Establishment of HUA rat model and administration

**Figure 1:**
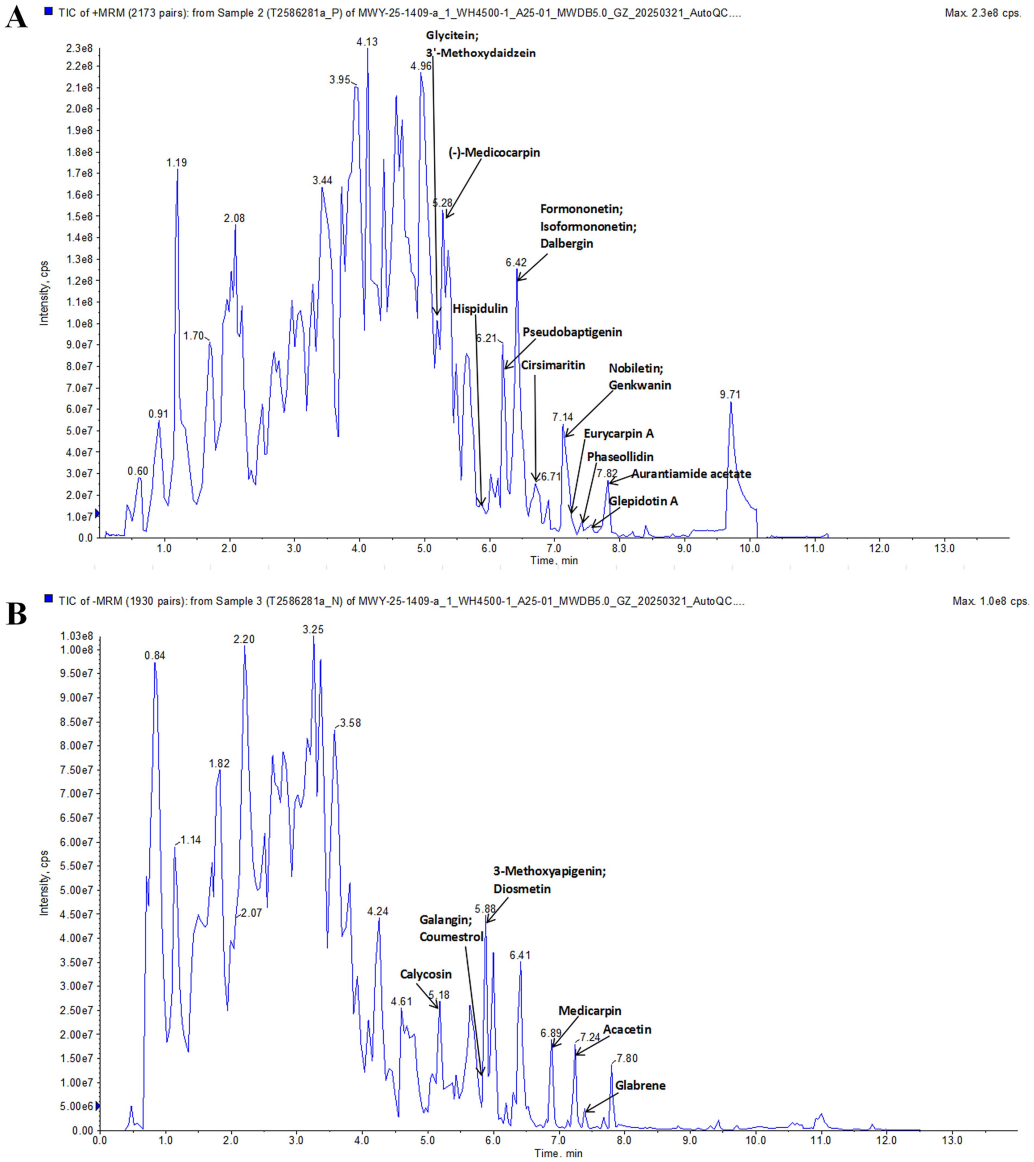
UPLC-ESI-MS/MS analysis of PLR. **(A)** Positive ion mode. **(B)** Negative ion mode.

Six-week-old, specific pathogen-free (SPF) male Sprague–Dawley (SD) rats were purchased from Liaoning Changsheng Biotechnology Co., Ltd. (Benxi, China), and housed in Dalian Medical University Laboratory Animal Center under standard environmental conditions, with ad libitum access to sterile water and food. The experimental protocols adhered to the ethical guidelines set forth by the Ethics Committee of Dalian Medical University (No. AEE21053). Following a one-week acclimatization period, the rats were randomly divided into five groups, each containing of six rats: (1) the normal control group (NC), (2) the model group (M), (3) an allopurinol-treated positive control group (PC), (4) the high-dose PLR group (PLR-H), and (5) the low-dose PLR group (PLR-L). The hyperuricemic rat model was established by administering potassium oxonate via gavage (31, 32) at a dose of 1,000 mg/kg to all groups except NC group. After a 6-h interval, rats in the PC group, PLR-H group, and PLR-L group were administered oral gavage with allopurinol (27 mg/kg) or PLR [0.4 g/kg and 0.2 g/kg, respectively. Equivalent to a human daily dosage of 4 g and 2 g (33)] at a volume of 1 mL per 100 g body weight. In contrast, rats in the NC group and M group received an equal volume of distilled water via oral gavage. The study spanned a total of 28 days, with the experimental design illustrated in Figure 1A. Weekly body weight measurements were taken, and serum uric acid levels were measured biweekly throughout the experiment, where serum was collected from the inner canthus of the eyes of rats anesthetized with isoflurane. On the final day of treatment, rats were euthanized via abdominal aorta blood collection under anesthesia induced by 20% urethane (0.5 mL/100 g body weight), and samples of liver, kidney, spleen, and colonic contents were collected and preserved at −80 °C for subsequent analysis.

#### 2.4.2 Biochemical parameter detection

The collected blood samples were centrifuged at 4 °C and 3,000 g for 15 min, The resulting serum supernatant was subsequently utilized for the quantitative analysis of serum UA, BUN, CRE, ADA and XOD, following the manufacturer’s instructions. Hepatic XOD activity was evaluated using an XOD activity assay kit.

#### 2.4.3 ELISA analysis

Serum samples were utilized for ELISA tests. The concentrations of AST, ALT, TNF-*α*, IL-6, IL-1*β*, NF-κB were measured in strict accordance with the manufacturers’ protocols.

#### 2.4.4 Histopathologic analysis of kidney and liver

Rat liver and kidney tissues were fixed in 4% paraformaldehyde. Subsequently, the fixed tissues underwent a series of processing steps, including dehydration, embedding, sectioning, and hematoxylin–eosin (H-E) staining. The histomorphological changes such as edema, degeneration, necrosis, hyperplasia, fibrosis, and inflammatory changes were meticulously observed under microscope.

### 2.5 16S rRNA sequencing

Initially, DNA was extracted from the samples using the CTAB method (34), following the manufacturer’s instructions. Subsequently, PCR amplification was conducted. The PCR products were purified and quantified using AMPure XT beads (Beckman Coulter Genomics, Danvers, MA, United States) and Qubit (Invitrogen, United States), respectively. The purified PCR products were then evaluated with the library quantification kit of the Agilent 2,100 Bioanalyzer (Agilent, United States) and Illumina (Kapa Biosciences, Woburn, MA, United States). Duplex sequencing with a read length of 2 × 250 bp was performed on a NovaSeq 6,000 sequencer. Finally, the raw sequencing data were processed by splitting to remove barcodes and primer sequences, followed by splicing, filtering, and DADA2 denoising. Alpha diversity and beta diversity analyses were then conducted based on the derived ASV feature sequences and ASV abundance table. Species annotation was performed using the SILVA database (Release 138, https://www.arbsilva.de/documentation/release138/) and the NT-16 s annotation database.

### 2.6 Data mining for network pharmacology

The chemical components of PLR were retrieved from multiple online databases, such as CNKI^2^, PubMed^3^, TCMSP^4^, TCMID^5^, BATMAN^6^, which was integrated with the results of UPLC-MS analysis. Subsequently, the PubChem^7^ platform was used to obtain the SMILES number for the identified components, which were then subjected to target prediction using the Swiss Target Prediction platform.^8^ Using “Hyperuricemia” as the keyword, a comprehensive retrieval of disease-associated targets was executed across multiple authoritative databases: GeneCards^9^, Drugbank^10^, and OMIM.^11^ The Venny 2.1.0 platform^12^ was employed to identify the intersection targets between the disease and components. Protein – protein interaction (PPI) analysis was carried out using the STRING database^13^, and the results were visualized with Cytoscape software.

For the core targets, GO and KEGG pathway enrichment analyses were conducted using the DAVID database^14^ (35), with a significance level set at *p* < 0.05. The top 10 signaling pathways were visualized using a micro—information platform.^15^ The outcomes highlighted the top 10 significantly implicated signaling pathways, which were vividly depicted through a micro-information platform, offering insights into the molecular mechanisms underpinning the therapeutic potential of PLR in hyperuricemia management.

The predicted key target proteins were validated using quantitative real-time PCR (qRT-PCR) analysis. Total RNA was extracted from the kidney tissue samples from rats utilizing an RNA extraction kit. The concentration and purity of the extracted RNA were measured with a Nanodrop 2000 spectrophotometer. Subsequently, the RNA was subjected to reverse transcription followed by quantitative amplification. The relative gene expression levels were calculated using the ΔΔCT method. The primer sequences for the target genes are detailed in Supplementary Table S1.

Molecular docking was further employed to evaluate the binding affinity of key compounds with the core target (36). The crystal structures of the proteins were obtained from the RCSB PDB database^16^, while the chemical structures of the compounds were retrieved from the PubChem database (see text footnote 7). Proteins and compounds preparation were conducted using PyMOL (The PyMOL Molecular Graphics System, Version 2.6.2 Schrödinger, LLC) and ChemBio3D Ultra 14.0.0.117 respectively, including the removal of non-protein molecules (e.g., water, bound ligands). The processed structures were then converted into PDBQT format using AutoDock Tools 1.5.7. A docking grid box was subsequently drawn using AutoDock Tools 1.5.7 with maximum spacing to ensure that the binding pocket encompassed the majority of the protein. Finally, molecular docking was performed using AutoDock Vina 1.1.2, and binding energies were calculated. A binding energy of less than or equal to −5.5 kcal/mol was set to define strong binding interactions. The docking results were visualized using PyMOL.

### 2.7 Statistical analysis

All experimental data were analyzed using SPSS 20.0 and GraphPad Prism 10.1.2. Differences between groups were determined by one-way ANOVA followed by an independent Student’s *t*-test or Dunnett’s T3 test. The data are presented as mean ± standard deviation (SD), with *p* < 0.05 considered statistically significant.

## 3 Results

### 3.1 Chemical component analysis of PLR aqueous extract

UPLC-ESI-MS/MS was used to analyze the components of PLR. Figure 1 shows the total ion chromatography in positive and negative ion mode. By using the Metware database, 23 compounds were identified, including (−)-Medicocarpin, Formononetin, Hispidulin, Glycitein, Genkwanin, Cirsimaritin, Aurantiamide acetate, 3′-Methoxydaidzein, Isoformononetin, Dalbergin, Glepidotin A, Phaseollidin, Pseudobaptigenin, Nobiletin, Eurycarpin A, Galangin, Acacetin, Calycosin, 3-Methoxyapigenin, Glabrene, Diosmetin, Medicarpin, Coumestrol. These compounds are listed in Supplementary Table S2.

### 3.2 PLR promote weight gain and alleviates the SUA in HUA rats

**Figure 2:**
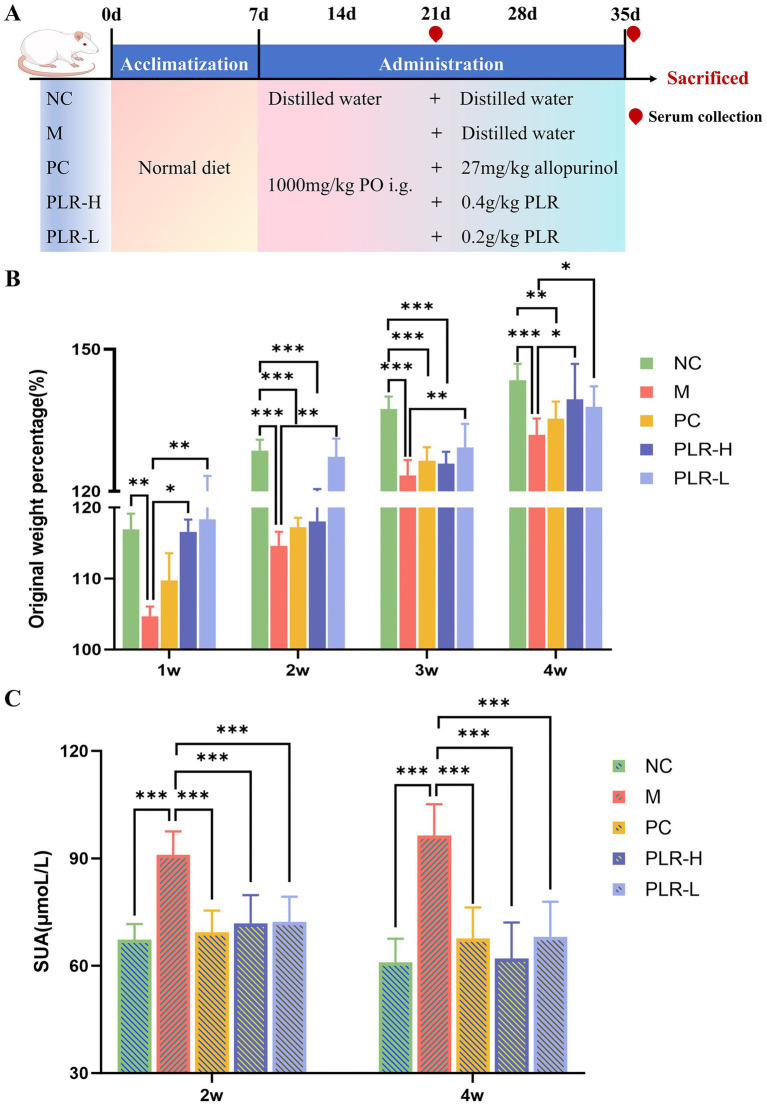
The effect PLR on the weight gain and uric acid levels in HUA rats. **(A)** The experimental design and administration scheme. **(B)** Original weight percentage among groups. **(C)** uric acid levels among groups at 2 weeks and the end of the fourth week. All the data are presented as the mean ± SD, Statistical significance was denoted as ^##^ *P*<0.01, ^###^ *P*<0.001 comparing with the control group, and ^*^ *P*<0.05, ^**^ *P*<0.01, ^***^ *P*<0.001 comparing with the model group. NC: the normal control group; M: the model group; PC: an allopurinol-treated positive control group; PLR-H: the high-dose PLR group; PLR-L: the low-dose PLR group, the abbreviations for groups are consistent across all subsequent figures.

The animal experiment was conducted to investigate the potential efficacy of PLR in HUA rats, with the experimental design and administration scheme shown in Figure 2A. As illustrated in the bar chart of body weight (Figure 2B), the weight of rats in all groups exhibited an increasing trend throughout the experiment, which indicates that the modeling and drug administration procedures did not cause significant damage to the rats. However, compared with the control group, the original weight percentage in the model group significantly decreased (*P*<0.01, *P*<0.001). In contrast, both the high-dose and low-dose of PLR significantly promoted weight gain in the model rats (*P*<0.05, *P*<0.01). These preliminary results indicate that PLR exerts a positive regulatory effect on HUA rats. Furthermore, the subsequent therapeutic effects of PLR, such as serum uric acid reduction can be attributed to its specific therapeutic action against HUA, rather than non-specific improvement in overall animal health. Additionally, these findings also provide valuable insights for optimizing modeling and dosing administration in future experiments. Two weeks and at the end of the fourth week of model establishment and drug administration, the serum was collected from each group of rats to measure the UA level. The results are presented in Figure 2C. Compared with the control group, UA levels in the model group were significantly elevated (*P*<0.001), confirming the successful establishment of the HUA model. Moreover, allopurinol, as well as both the high-dose and low-dose of PLR, could significantly reduce the UA levels (*P*<0.001). At the end of the experiment, the high-dose PLR demonstrated the most significant effect in reducing uric acid levels. These results indicate that PLR exhibits a uric acid-lowering effect and follows a certain dose-dependent relationship.

### 3.3 PLR reduce the activity of UA synthetase in HUA rats

**Figure 3:**
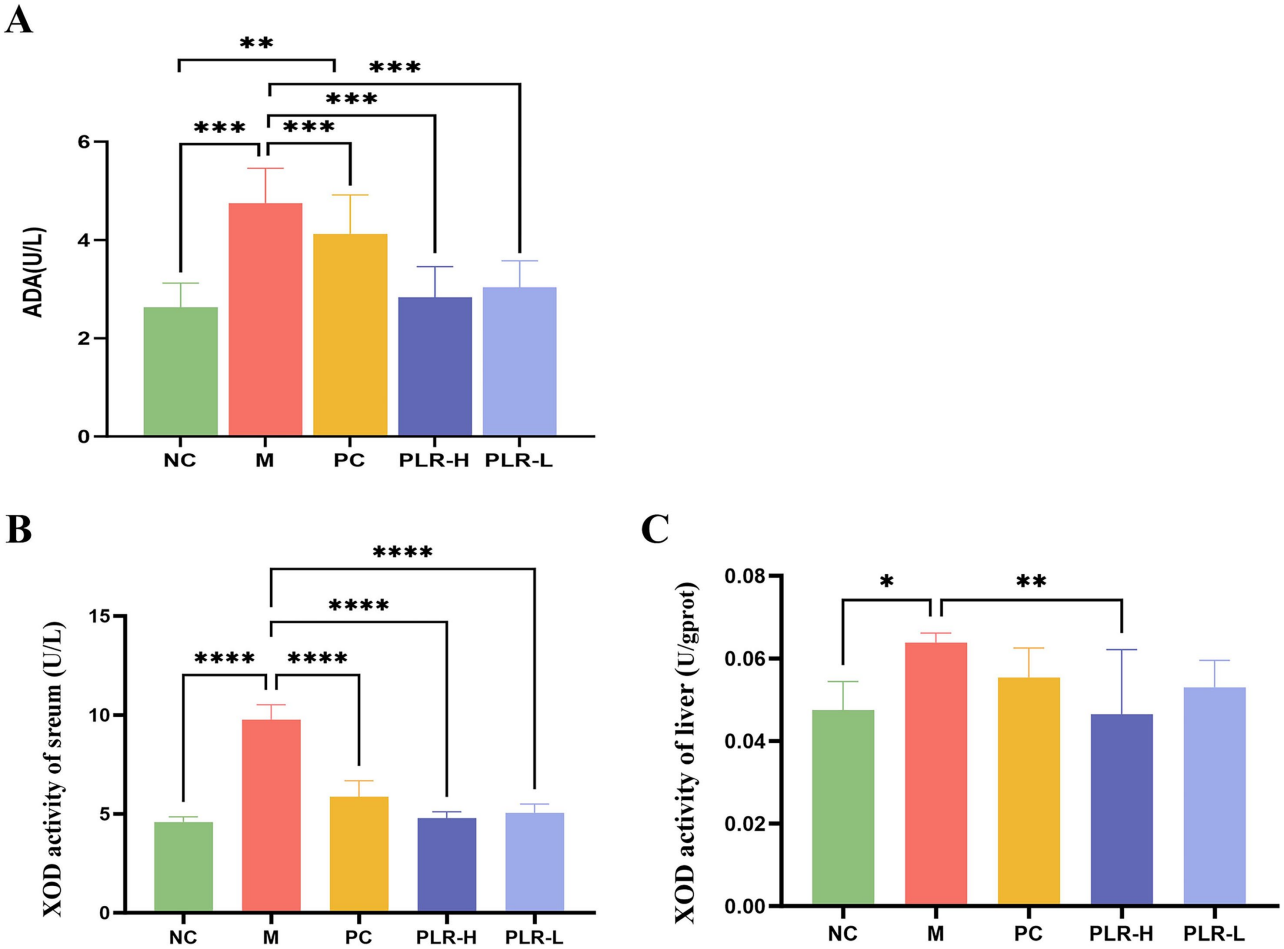
The effect PLR on the UA synthetase in HUA rats. **(A)** The activity of ADA among groups. **(B)** The activity of XOD in serum among groups. **(C)** The activity of XOD in liver among groups. All the data are presented as the mean ± SD, Statistical significance was denoted as ^##^ *P*<0.01, ^###^ *P*<0.001 comparing with the control group, and ^***^ *P*<0.001 comparing with the model group.

Adenosine deaminase (ADA) and xanthine oxidase (XOD) are pivotal enzymes in the uric acid synthesis pathway. ADA catalyzes the conversion of adenine nucleosides to hypoxanthine, which is subsequently oxidized to xanthine by XOD, and ultimately converted to uric acid. Consequently, elevated activities of both ADA and XOD are associated with increased UA production. Given that PLR effectively alleviated UA level, we next investigated the regulatory effects of PLR on UA synthetase. As shown in Figures 3A–C, compared with the control group, the model group exhibited significantly increased activities of ADA and XOD (*P*<0.001). In contrast, both the XOD-inhibitor allopurinol group and the PLR groups significantly suppressed ADA and XOD activities (*P*<0.01, *P*<0.001). The PLR-H group showed better effects than the PLR-L group. These results indicated that PLR has inhibitory activity against uric acid synthetase and prevent the over production of UA in HUA rats (Figure 3C).

### 3.4 PLR alleviates the expression of inflammatory factors in HUA rats

**Figure 4:**
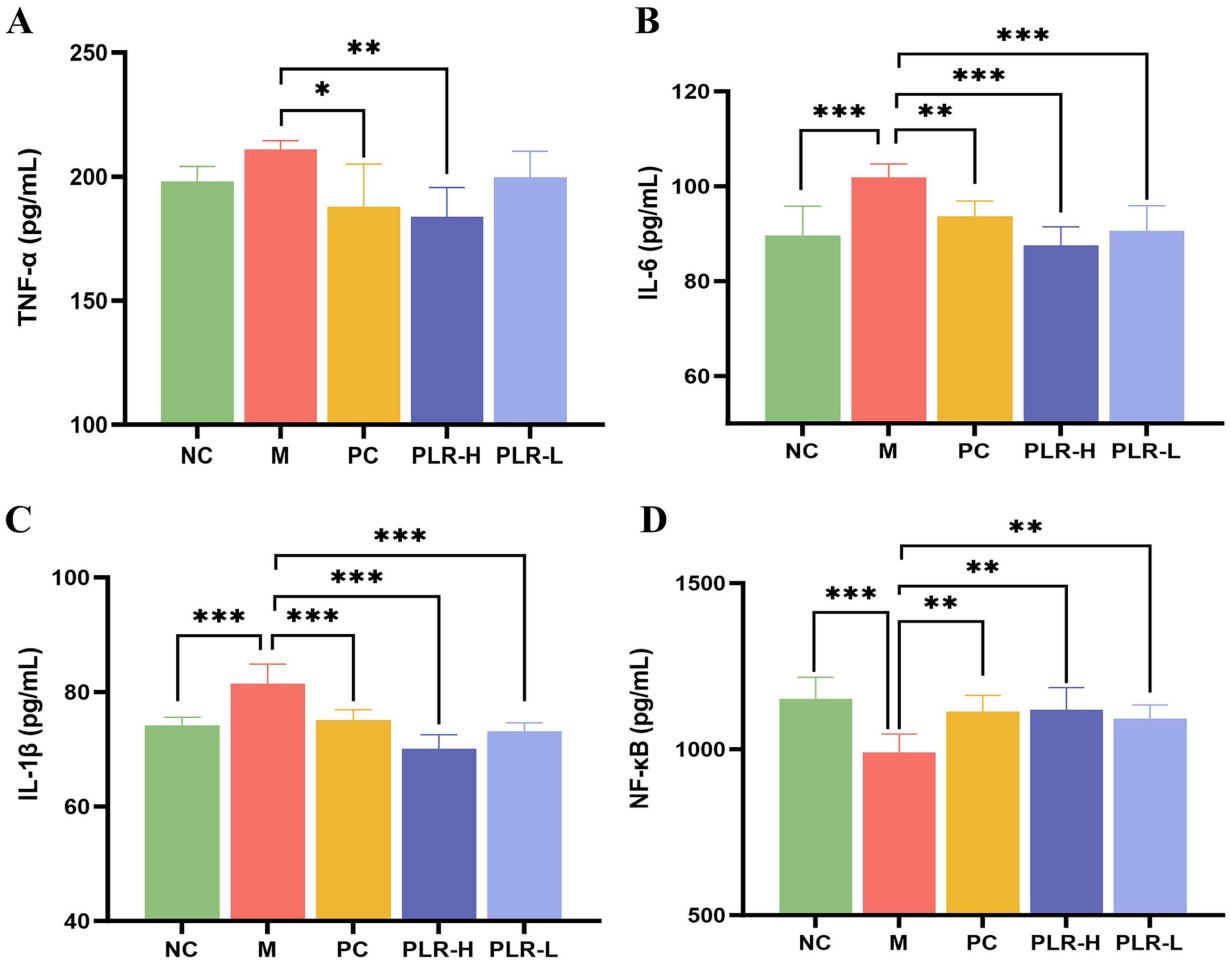
The effect PLR on the expression of inflammatory factors in HUA rats. **(A)** The expression level of TNF-*α* among groups. **(B)** The expression level of IL-6 among groups. **(C)** The expression level of IL-1*β* among groups. **(D)** The expression level of NF-κB among groups. All the data are presented as the mean ± SD, Statistical significance was denoted as ^###^*P*<0.001 comparing with the control group, and ^*^ *P*<0.05, ^**^ *P*<0.01, ^***^ *P*<0.001 comparing with the model group.

Elevated uric acid levels can activate signaling pathways such as NF-κB, NLRP3 and TLRs, increase the production of reactive oxygen species (ROS), and impair intestinal barrier function. These effects promote the release of inflammatory factors and contribute to the formation of hyperuricemia. ELISA kits were then used to measure the levels of inflammation levels in serum of HUA rats. The results for the levels of TNF-*α*, IL-6, IL-1*β*, and NF-κB were shown in Figures 4A–D. Compared with the control group, the model group showed significantly elevated levels of IL-6 and IL-1*β* (*P*<0.001), along with increased TNF-α and a marked reduction in NF-κB (*P*<0.001). Following drug administration, these alterations were significantly attenuated (*P*<0.05, *P*<0.01, *P*<0.001), with the PLR-H group exhibiting the most pronounced effect. Notably, while existing literature suggests that NF-κB expression generally correlates with cytokine levels, our findings indicate that cytokine production in HUA rats may not be completely dependent on NF-κB regulation. An alternative explanation involves negative feedback: persistent NF-κB activation can induce powerful feedback regulators like IκBα and A20, thereby suppressing its own activity and subsequent cytokine production. Importantly, PLR appears to restore the physiological regulatory mechanism.

### 3.5 PLR exhibits no hepatotoxicity and alleviates renal function in HUA rats

**Figure 5:**
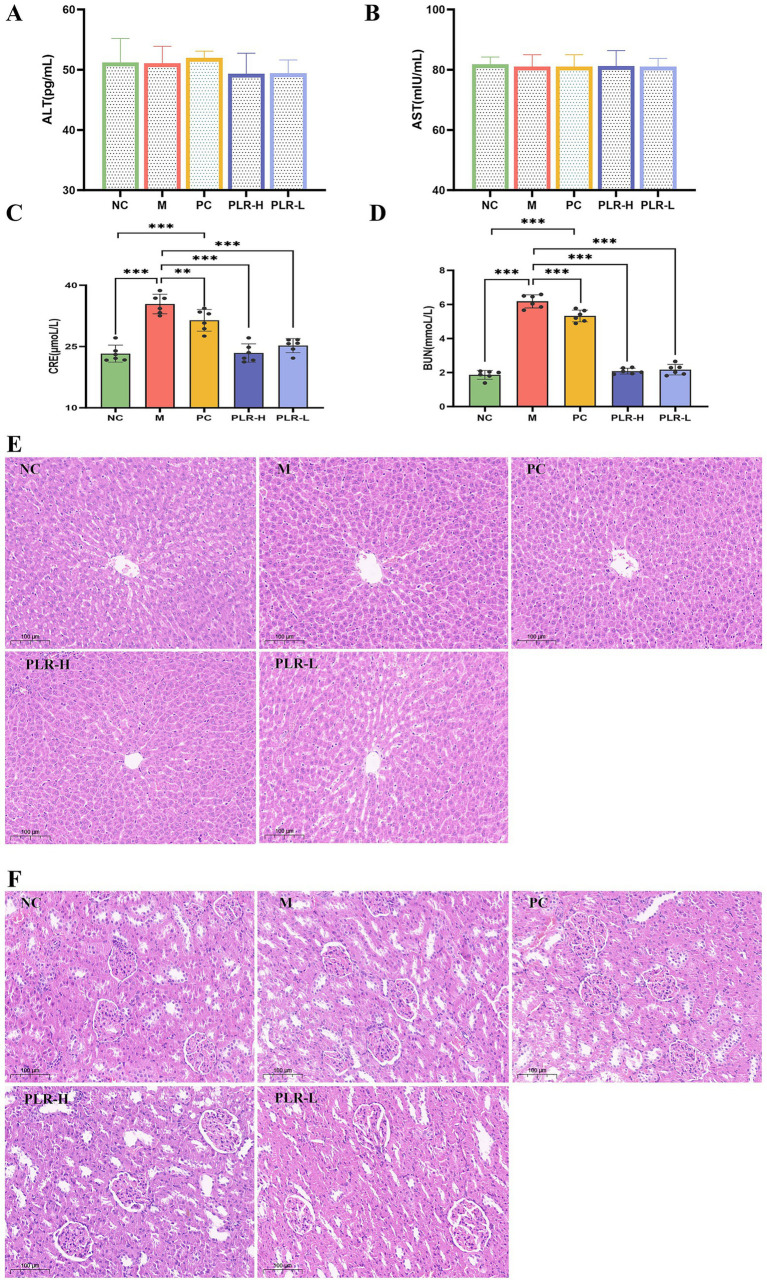
The effect PLR on the hepatic and renal function in HUA rats. **(A)** The expression level of ALT among groups. **(B)** The expression level of AST among groups. **(C)** The expression level of BUN among groups. **(D)** The expression level of Cre among groups. **(E)** H-E staining of kidney sections among groups. **(F)** H-E staining of liver sections among groups. All the data are presented as the mean ± SD, Statistical significance was denoted as ^###^*P*<0.001 comparing with the control group, and ^**^P<0.01, ^***^P<0.001 comparing with the model group.

The liver and kidneys are the primary organs responsible for uric acid synthesis and excretion. Under conditions of hyperuricemia, both hepatic and renal functions may be affected. Serum BUN and Cre levels serve as key indicators of renal function, while abnormally elevated AST and ALT levels are common markers of liver injury. The results of our research are shown in Figure 5, the levels of AST and ALT among groups showed no significant difference (Figures 5A,B), indicating that the potassium oxazenate-induced hyperuricemia model did not cause obvious liver injury in rats and that the tested drug PLR exhibited good safety. The levels of BUN and Cre in model group were significantly higher than those in the control group (*P*<0.001), confirming the association between hyperuricemia and renal injury. Following PLR treatment, BUN and Cre levels in both low-dose (PLR-L) and high-dose (PLR-H) groups were significantly reduced toward control levels (*P*<0.01, *P*<0.001), and the effects were superior to those of the allopurinol group (Figures 5C,D).

The above results initially indicate that there is certain renal injury in HUA rats, and that PLR exerts a renal protective effect. However, further in-depth analysis via histopathological examination is necessary to validate these findings. The HE staining results revealed that the pathological structure of liver in each group of rats was essentially normal, with no obvious inflammatory cell infiltration or hepatocyte edema observed (Figure 5E). In renal tissues of each group, no significant pathological abnormalities were detected, instead, glomerular and tubular structures remained intact, and tubular epithelial cells were orderly arranged (Figure 5F).

These results are inconsistent with the elevation of BUN and Cre levels. This discrepancy may be attributed to the relatively short model establishment duration and moderate severity of the model, which may not have induced pathological structural damage. Additionally, the tested agent PLR was medicinal and edible raw materials administered at relatively low doses. This model also more closely meets the requirements for asymptomatic hyperuricemia and preventive medication. These findings demonstrate that PLR exerts a certain protective effect on the kidneys and does not induce hepatotoxicity.

### 3.6 PLR alleviates the intestinal flora in HUA rats

**Figure 6:**
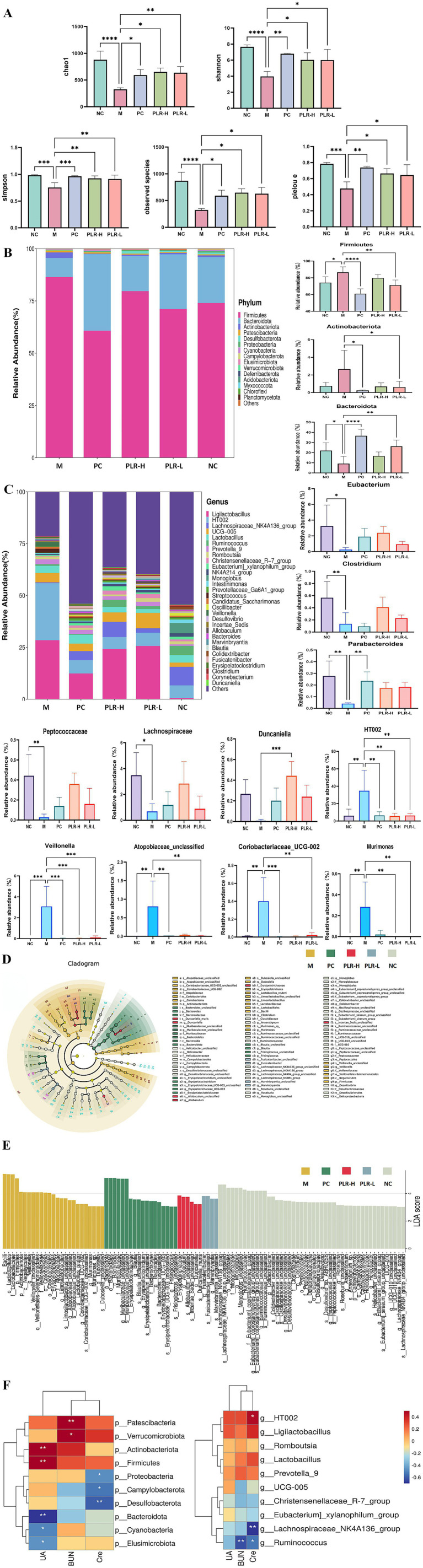
The dysregulation effect of PLR on the intestinal flora of HUA rats. **(A)** Alpha diversity analysis, including chao 1, Shannon, Simpson, observed species, and pielou e. **(B)** Relative abundance analysis at phylum level and the intestinal flora exhibited significant differences in abundance. **(C)** Relative abundance analysis at genus level and the intestinal flora exhibited significant differences in abundance. **(D,E)** Effect size (LEfSe) analysis and barplot illustrating the significant differences in intestinal flora among groups as determined by linear discriminant analysis (LDA). **(F)** Correlation analysis of biochemical indexes with intestinal flora at both phylum and genus levels. The darker the red color, the stronger the positive correlation; the darker the blue color, the stronger the negative correlation. The correlations are ^*^ *P*<0.05, ^**^ *P*<0.01. All the data are presented as the mean ± SD, Statistical significance was denoted as ^*^ *P*<0.05, ^**^ *P*<0.01, ^***^ *P*<0.001.

The intestine is another important pathway for uric acid excretion, apart from the kidneys. Indeed, emerging evidence in recent years indicates that dysbiosis of the intestinal flora is closely associated with the pathogenesis of HUA. To evaluate the effect of PLR on the intestinal flora of HUA rats, we conducted 16S rDNA sequencing of rats’ colon contents. Firstly, the results of the *α* diversity of intestinal flora are shown in Figure 6A. The chao1, Shannon, Simpson, observed species, and pielou e indices were significantly lower compared to the control group (*P*<0.01). Both allopurinol and PLR interventions significantly increased the values of these indices (*P*<0.05, *P*<0.01, *P*<0.001), indicating that both HUA and PLR interventions directly affect the abundance of the intestinal flora in rats.

To further elucidate the regulatory effect of PLR on the intestinal flora, we analyzed the abundance of intestinal flora at the phylum and genus levels. The results at the phylum level are shown in Figure 6B. The dominant microbiota primarily comprised Firmicutes, Bacteroidetes, Actinobacteria, Patescibacteria, Desulfobacteria, Proteobacteria and Cyanobacteria. Statistical analysis of the top three phyla (Firmicutes, Bacteroidetes, and Actinobacteria) revealed that compared with the control group, the abundances of Firmicutes was significantly increased (*P*<0.05), and Actinobacteria showed an upward trend in the model group, while Bacteroidetes abundance was significantly decreased (*P*<0.05). Both the PLR-L and allopurinol groups could significantly regulate these changes (*P*<0.05). The PLR-H exhibited a regulatory trend but showed no significant difference from the model group. At the genus level, as shown in Figure 6C, HUA rats exhibited significant decreases in the relative abundance of Eubacterium, Clostridium, Parabacteroides, Peptococcaceae, Lachnospiraceae, and Duncaniella (*P*<0.05, *P*<0.01), while showing significant increases in the relative abundance of HT002, Veillonella, Atopobiaceae_unclassified, Coriobacteriaceae UCG-002, and Murimonas (*P*<0.001). PLR intervention reversed the abundance of these genera (*P*<0.05, *P*<0.01, *P*<0.001). Linear discriminant analysis (LDA) combined with effect size (LEfSe) analysis revealed that PLR modulated the intestinal flora from the phylum to genus levels (Figures 6D,E).

To explore the relationship between intestinal flora alterations and HUA remission, Spearman correlation analysis was employed to investigate the associations between bacteria and HUA-related biochemical variables at the phylum and genus level. The results (Figure 6F) showed that UA was positively associated with Actinobacteriota, Firmicutes (*P*<0.01), and negatively associated with Bacteroidota, Cyanobacteria, and Elusimicrobiota (*P*<0.05, *P*<0.01). BUN was positively associated with Patescibacteria, Verrucomicrobiota (*P*<0.05, *P*<0.01), and negatively associated with Ruminococcus (*P*<0.01). Cre was positively associated with HT002 (*P*<0.05), and negatively associated with Proteobacteria, Campylobacterota, Desulfobacterota, Lachnospiraceae NK4A136 group, and Ruminococcus (*P*<0.05, *P*<0.01).

These findings indicate that intestinal flora dysregulation occurs in HUA rats, and PLR may exert an anti-hyperuricemia effect by regulating these disturbed composition of gut microbiota. These results provide valuable insights into the treatment of HUA.

### 3.7 PLR exerts an anti-HUA effect via multi-pathway and multi-target based on the network pharmacological analysis and PCR, molecular docking verification

**Figure 7:**
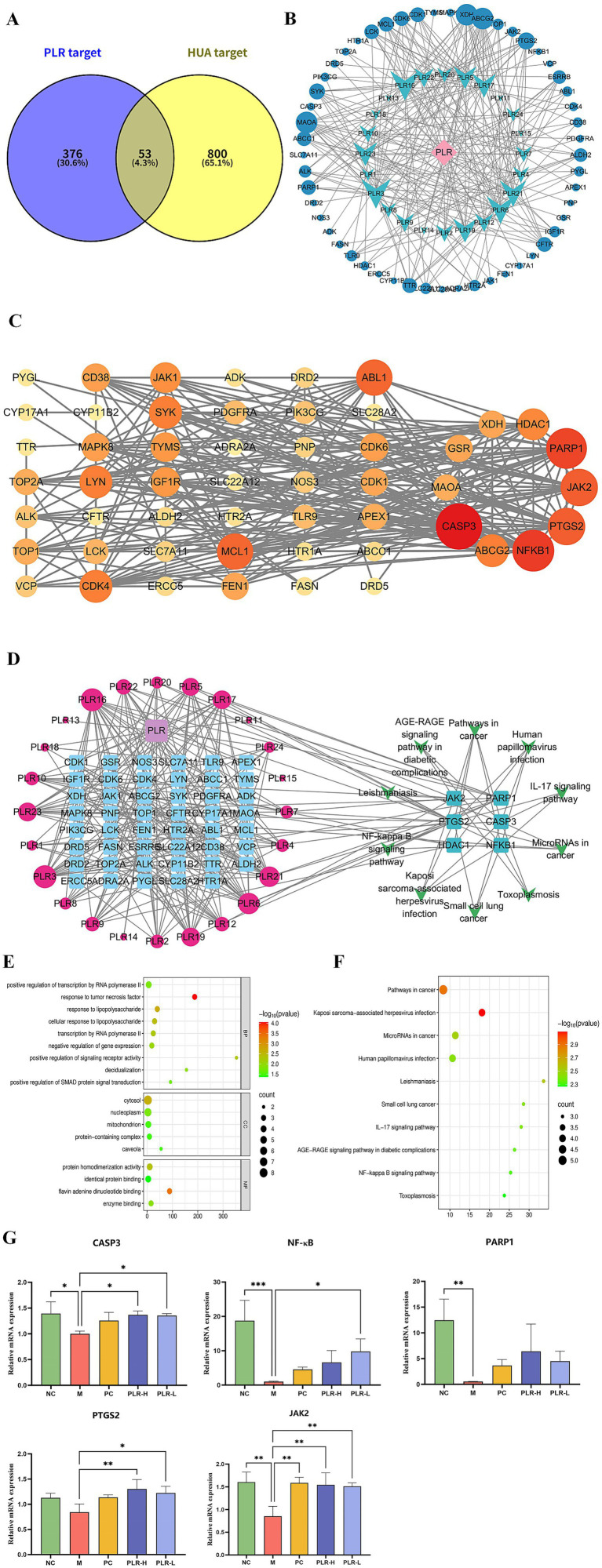
Network pharmacology analysis revealed the potential targets and pathways of PLR in the management of HUA. **(A)** Venn diagram of PLR components and disease targets. **(B)** The network of PLR components and their intersection targets in HUA. The blue circle represents the target, blue-green dovetail shapes represents the components of PLR. **(C)** Protein–protein interaction (PPI) network of HUA targets modulated by PLR. Each node corresponds to a protein, The intensity of the coloration, ranging toward red, signifies the heightened significance of the respective node. **(D)** Composition-target-pathway network. The interactions between components of PLR and targets are depicted on the left, where red circles denote the components of PLR and blue squares represent the targets. The size of the circles is proportional to the significance of the component’s role in mitigating hyperuricemia: larger circles indicate a more substantial contribution. On the right, the top six enriched pathways and their associated targets are illustrated. **(E)** GO enrichment analysis ranked the top 10 signaling pathways. **(F)** KEGG enrichment analysis ranked the top 10 signaling pathways. **(G)** Effects of PLR on the expression of the top three target protein genes. The data are presented as the mean ± SD, Statistical significance was denoted as ^*^ *P*<0.05, ^**^ *P*<0.01, ^***^ *P*<0.001 when compared to the model group, while ^#^ *P*<0.05, ^##^ *P*<0.01, ^###^*P*<0.001 indicated comparisons relative to the normal control group.

Despite the compelling evidence from our data demonstrating the protective effect of PLR in the hyperuricemia (HUA) rat model, the underlying mechanisms that confer these therapeutic benefits remain largely unclear. To clarify the active constituents and potential targets of PLR in HUA, a network pharmacological analysis was carried out. Consequently, a total of 24 potential active compounds (screened based on ADME-related criteria: oral bioavailability (OB) > 30%, drug-likeness (DL) > 0.18, and high gastrointestinal absorption) and 376 corresponding targets associated with PLR were identified, while 800 targets related to HUA were retrieved, among which 53 were found to overlap using the Venn tool (Figure 7A). Based on these overlapping targets, we constructed a network of PLR components and their intersection targets (Figure 7B). Subsequently, employing STRING and Cytoscape analyses, we obtained a total of 10 core targets, including CASP3, ABCG2, NFKB1, PTGS2, JAK2, PARP1, HDAC1, XDH, GSR, and MAOA (Figure 7C). GO and KEGG enrichment analyses were then performed. The results, presented in Figures 5E,F respectively, show the top 10 pathways. Pathways such as the pathways in cancer signaling pathway, NF-κB signaling pathway, and IL-17 signaling pathway were significantly enriched. Additionally, we constructed a composition-target-pathway network (Figures 7D–F). To validate the reliability of these findings, we used qRT-PCR to verify the expression levels of the top five core targets: CASP3, NF-κB, PARP1, PTGS2, and JAK2. As depicted in Figure 7G, the expression of the five core targets genes were significantly downregulated (*P*<0.05, *P*<0.01, *P*<0.001) in the HUA rat model. Administration of PLR significantly improved the expression of these genes (*P*<0.05, *P*<0.01), indicating its anti-HUA effects.

**Figure 8:**
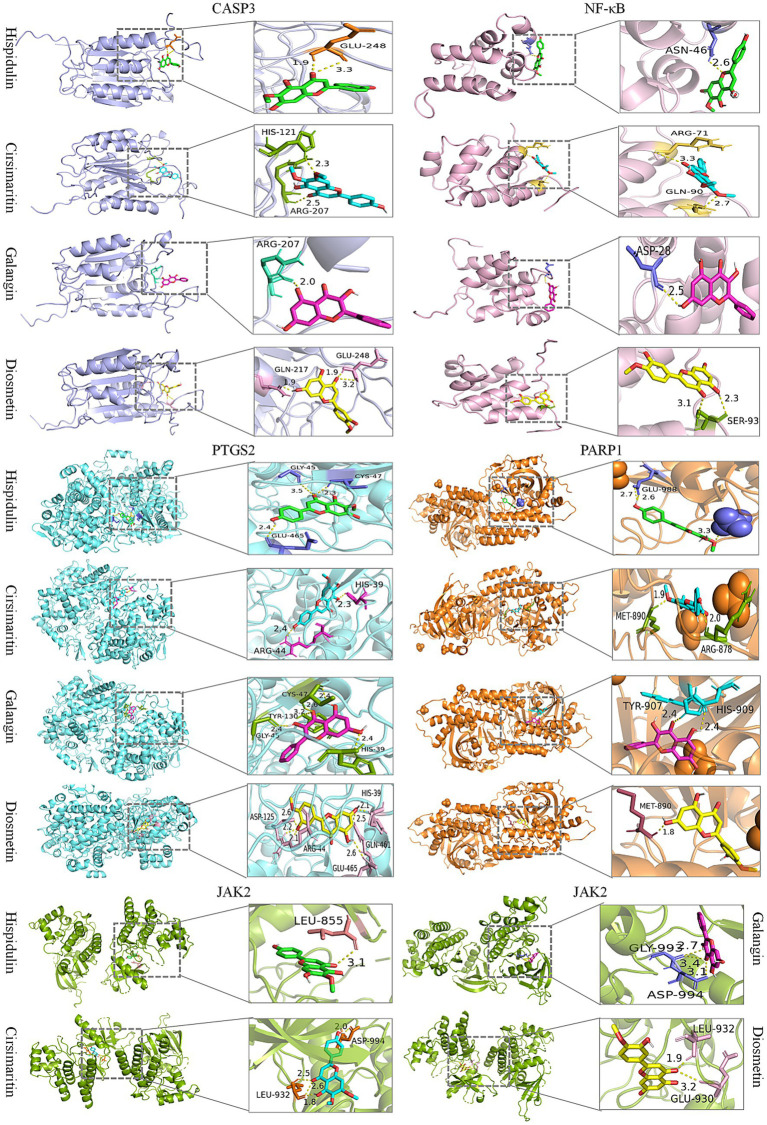
Molecular docking of candidate compounds (hispidulin, cirsimaritin, galangin, and diosmetin) with core targets of HUA (CASP3, NF-κB, PTGS2, PARP1, and JAK2).

We further employed molecular docking to verify the binding interactions between the top five core targets-CASP3 (PDB ID: 1GFW), NF-κB (PDB ID: 2DBF), PARP1 (PDB ID: 7KK2), PTGS2 (PDB ID: 5F19), and JAK2 (PDB ID: 2XA4), and the candidate compounds hispidulin, cirsimaritin, galangin, and diosmetin. As shown in Figure 8, all core targets formed stable binding with the four compound, exhibiting binding energies below −5.0 kcal/mol (Supplementary Table S3). The results suggest that the aforementioned compounds from PLR can modulate the critical targets associated with HUA and be and potentially contribute to the biological processes of uric acid lowering.

## 4 Discussion

Hyperuricemia (HUA), as a global health concern, is witnessing a rising prevalence annually, and current drug treatments are plagued by notable adverse effects. Consequently, the exploration of safer and more effective prevention and treatment approaches has become a pressing need (3). In this context, medicinal and edible Chinese medicine offers an alternative therapy for HUA. Numerous studies have demonstrated that medicinal food homologous plant can reduce uric acid levels. A wide range of plant species and used part, like Chinese quince, lemon, chicory, tea, Flos sophorae, lotus leaf, chrysanthemum flowers, Dendrobium officinale leaves, and Agrocybe aegerita, have been utilized in many countries such as China, Korea, India, and Brazil for extended periods (37, 38). Furthermore, flavonoids, alkaloids, polyphenols, phenolic acids, saponins, and other phytochemicals derived from natural sources have also exhibited beneficial properties in the prevention and treatment of HUA. The underlying mechanisms primarily involve targeting xanthine oxidase enzymes to inhibit uric acid production, modulating the activity of transport proteins to enhance uric acid excretion, or alleviating oxidative stress and inflammation (39). In the present study, we focused on *Puerariae lobatae* Radix (PLR), given its traditional applications in the management of metabolic diseases. However, there is a paucity of research specifically examining the individual effects and the underlying mechanisms of PLR aqueous extract against HUA. Our findings demonstrate that PLR exerts favorable preventive and therapeutic effects against hyperuricaemia. Mechanistically, this study moves beyond the established role of xanthine oxidase inhibition to reveal a more comprehensive mode of action. We show that PLR also facilitates uric acid excretion by modulating the gut microbiota and alleviating compensatory feedback mechanisms in renal tissue. Furthermore, expanding beyond the well-characterized active component puerarin, our research is the first to predict and preliminarily validate hispidulin, cirsimaritin, galangin, and diosmetin as potential key active constituents contributing to PLR’s UA-lowering effect. This finding expands the current understanding of the material basis underlying PLR’s anti-HUA efficacy, providing new insights into the multi-component synergy of PLR.

Potassium oxonate (PO), a triazene compound structurally analogous to the purine ring of urate. It can competitively bind to uricase, thus blocking the degradation of uric acid to allantoin and increasing the uric acid level. Commonly, it serves as a modeling agent for establishing HUA animal models (14). Given that the gene encoding uricase has been inactivated during human evolution (29), this modeling approach can largely simulate the pathogenesis of human hyperuricemia. In our study, PO was administered orally at a dose of 1,000 mg/kg to induce an asymptomatic hyperuricemia (AH) rat model, in accordance with previously published reports (32). The results demonstrated that the serum uric acid (SUA) levels in the model rats were significantly elevated compared to those in the control group. Moreover, no overt symptoms such as arthritis were observed, indicating the successful establishment of an AH model. Both high and low doses of PLR administration have been observed to decrease SUA levels, demonstrating its efficacy as an anti-HUA agent.

UA is the terminal metabolite of purine metabolism, which is primarily derived from the degradation of nucleic acids and other purine compounds via cellular metabolic processes, as well as from dietary purines (40). UA homeostasis is meticulously regulated by a coordinated interplay of multiple organs within the body, and encompasses a variety of enzymes and complex biochemical processes. Shortly, adenosine (ADA) in the liver catalyzes the degradation of adenine nucleoside to hypoxanthine nucleoside, which is then converted to hypoxanthine under the action of nucleoside phosphorylase. Hypoxanthine is subsequently oxidized to xanthine by xanthine oxidase (XOD), and xanthine is further oxidized to UA. Following the formation of UA, approximately one-third is excreted via the gastrointestinal tract, while two-thirds are eliminated through renal excretion (41). Therefore, ADA and XOD play crucial roles in the UA synthesis pathway, and the pivotal functions render them attractive targets for therapeutic interventions designed to reduce uric UA levels (42). In the present study, our results demonstrated that both high and low doses of PLR not only significantly inhibited the activity of serum ADA but also reduced the activity of liver XOD. This effect was superior to that of allopurinol, a well—known XOD inhibitor and the positive control in this study. These findings suggest that PLR lowers UA levels by decreasing uric acid production through its dual inhibitory effects on ADA and XOD.

The accumulation of UA in the body primarily hinges on hepatic production and renal as well as intestinal excretion. An elevated UA concentration can thus augment the burden on both the liver and kidneys, with the kidneys being particularly affected as approximately two—thirds of uric acid is excreted through them. This can lead to significant renal damage and even progress to chronic kidney disease (43). Clinically, serum blood urea nitrogen (BUN) and creatinine (CRE) levels are commonly used clinical indicators for evaluating renal function. With their elevation often reflecting severe renal insufficiency (44). An increase in these levels often indicates severe renal insufficiency. Additionally, abnormal changes in serum aspartate aminotransferase (AST) and alanine aminotransferase (ALT) levels are often associated with hepatic damage (45). In this study, the serum BUN and CRE levels in the model rats were significantly higher than those in the control group, there was no significant difference in serum AST and ALT levels among all groups, and histopathological analysis of rat liver and kidney performed using H&E staining had no obvious abnormality was found. The results indicated that under the experimental modeling conditions, the hyperuricemic rats exhibited a certain degree of kidney damage, yet no pathological alterations were observed. As a medicinal and edible material, it has been reported that high doses of PLR water extract (25 g/kg) is relatively safe for SD rats (46). In this study, PLR was administered at a relatively low dosage (equivalent to the dosages of 2 g and 4 g, respectively, for human administration), and significantly reduce the levels of BUN and CRE. These results suggest that PLR has no toxic effect and exerts certain nephroprotective effects, which is consistent with previous reports (47). However, the long-term safety profile of PLR and its precise effective dosage for hyperuricaemia treatment require further elucidation. Future studies should systematically investigate these aspects by incorporating at least three dose groups to establish a robust dose–response relationship, alongside a test substance-only control group (administering PLR to normal, non-modeled animals). This control is essential to distinguish whether the therapeutic effect arises from correcting the pathological state or altering normal physiology.

The intestine serves as a crucial potential organ for uric acid excretion, complementing the kidneys. Approximately one-third of urinary uric acid is excreted through the action of uric acid transporters and the metabolic activity of intestinal microflora (48). Extensive researches have demonstrated that the intestinal flora plays a significant role in regulating hyperuricemia. This association is primarily mediated through three ways: participating in purine metabolism, decomposing uric acid, and reducing uric acid concentrations; facilitating the excretion of uric acid via metabolites of intestinal microflora; and alleviating inflammation by repairing the intestinal barrier (49, 50). Therefore, regulating gut flora abundance is expected to be a promising strategy for the treatment and prevention of hyperuricemia. In this study, we utilized 16S rDNA sequencing to investigate the potential effects of PLR on the intestinal flora of hyperuricemia—model rats. As the results shown HUA induced significant alterations in the intestinal flora. At the phylum level, the abundance of Firmicutes and Actinobacteriota was significantly increased, while the abundance of Bacteroidota was significantly decreased in the model rats. These findings are consistent with previous studies (51). At the genus level, significant decreases were observed in the relative abundance of Eubacterium, Clostridium, Peptococcaceae, Parabacteroides, Lachnospiraceae, and Duncaniella, whereas significant increases were noted in the relative abundance of HT002, Veillonella, Atopobiaceae_unclassified, Coriobacteriaceae_UCG-002, and Murimonas. These changes were partially alleviated by the intervention of PLR.

It has been reported Eubacterium (52), Clostridium (53), Lachnospiraceae (54), Peptococcaceae (55) can produce butyrate and other short-chain fatty acid (SCFA), which plays a key role in immune regulation and intestinal inflammation inhibition. Parabacteroides have a close relationship with host health by regulating immunity, relieving inflammation and secreting metabolites, with the physiological characteristics of carbohydrate metabolism and secreting short-chain fatty acids (56). Duncaniella belongs to the Muribaculaceae family, a potential probiotic bacterial family, which can produce short-chain fatty acids, and has an active effect on regulating intestinal barrier, metabolic disorders and immune inflammation (57). Veillonella is a inflammatory pathogenic bacteria, which has a highly positive correlation with the concentrations of IL-1*β*, IL-6 and TNF-*α* (58). *Veillonella parvula* metabolizes immunosuppressive thiopurine drugs through xdhA xanthine dehydrogenase (59). In patients with elevated uric acid coronary heart disease complicated with nonalcoholic fatty liver disease, the abundance of Veillonella increased and the abundance of Parabacteroides reduced (60). While the reports had proved HT002 (61), Atopobiaceae (62), Coriobacteriaceae UCG-002 (63) and Murimonas (64) shown a positive correlation with pro-inflammatory cytokines, lipid metabolism, and the intestinal permeability. The intervention of PLR on HUA indicate that it can promote uric acid excretion through intestinal metabolites and improve inflammation by increasing the abundance of beneficial bacteria and decreasing the abundance of harmful bacteria.

Similar to other traditional Chinese medicines, PLR exhibits a complex and diverse profile of chemical constituents (20). This inherent property poses challenges to the exploration of its active ingredients and the corresponding mechanisms of efficacy. The integrated research strategy combining ultra—high—performance liquid chromatography/mass spectrometry (UHPLC/MS) with network pharmacology offers a novel perspective for addressing this issue (65). In this study, we utilized UPLC-MS to comprehensively analyze the chemical composition of PLR. Subsequently, network pharmacology was applied to investigate the potential targets and signaling pathways associated with the active ingredients. As a result, 23 main compounds in PLR were identified by UPLC-MS. Combined with the Traditional Chinese Medicine Systems Pharmacology (TCMSP) database, 24 active ingredients were determined for the network pharmacology analysis. Eventually, four components with the most therapeutic targets in HUA were predicted: hispidulin, cirsimaritin, galangin, and diosmetin. Moreover, five core targets of PLR for HUA treatment, such as CASP3, NF-κB, PTGS2, PARP1 and JAK2 were initially screened. Hispidulin is a naturally occurring flavonoid, which has an excellent anti-inflammatory effect (66). In uric acid nephropathy rat model, hispidulin can inhibit the release of IL-1*β*, IL-8, TNF-α, and IL-6, and intercept the activation of NF-κB signaling, thus effectively improve renal function injury (67). Additionally, hispidulin ameliorates endotoxin-induced kidney injury by suppressing inflammation, oxidative stress, and tubular cell death mediated by caspase-3 pathway (68). Hispidulin also can suppress the expressions of PTGS2 and NLRP3 inflammasome to improve cyclophosphamide-induced cystitis (69). Meanwhile, studies have reported that Hispidulin exerted xanthine oxidase inhibitory activity (70). Cirsimaritin can inhibit NF-κB nuclear translocation and NLRP3 inflammasome activation, and then inhibit maturation and release of IL-1*β* (71), it has an excellent anti-inflammatory activity (72). Galangin has demonstrated the capacity to inhibit xanthine oxidase (XO) activity (73). Galangin exerted an improvement on HUA by ameliorating gut-kidney axis dysfunction (74), and alleviate hyperuricemic nephropathy through regulating metabolic profiles and the JAK2/STAT3 signaling pathway (75). In uric acid treated tubular epithelial cells, galangin can suppress renal inflammation by the inhibition of NF-κB, PI3K/AKT and NLRP3 (76). Diosmetin is also an XO inhibitor (77), which can decrease UA production in hepatocytes (78), and has the potential to reduce inflammation (79). These studies suggest that hispidulin, cirsimaritin, galangin, and diosmetin are likely the key bioactive components in PLR responsible for its anti-hyperuricemic effects. Future work will involve *in vitro* enzymatic inhibition assays and *in vivo* pharmacodynamic studies to directly compare the efficacy of these components and experimentally validate their relative contributions to the observed uric acid-lowering effects.

We further used qRT-PCR to verify the mRNA expression of the five identified core targets in rat renal tissues. Notably, PLR reversed the downregulated expression of these genes in HUA rats, whereas these proteins are typically thought to be highly expressed in disease states. The molecular docking results further demonstrate strong binding interactions between these five target proteins and the four active compounds in PLR, with the average binding energy being less than −7 kcal/mol. It is well known that Caspase-3 (CASP3) is an executioner of cell apoptosis, however, accumulating evidence indicates that its activity can also affect the survival, proliferation, and differentiation of normal cells and tissues through both “nonautonomous” and “cell autonomous” mechanisms (80). NF-κB is one of the most important transcription factors, which plays critical roles in multiple physiological and pathological processes. Feedback inhibition arising from pathway activation is critical for normal homeostasis and operates at all levels in the pathway, from ligand-activated receptors to NF-κB gene transcription itself like IκBα, A20, and p105 (81, 82). In the present study, the reduced expression levels of NF-κB in the serum and renal tissues of the HUA model group may be attributed to the following mechanism: the modeling stimuli induced excessive activation of the NF-κB signaling pathway, which in turn triggered a robust negative feedback inhibition mechanism (e.g., sustained overexpression of IκBα and A20 proteins). Ultimately, this process caused the pathway to enter a state of “exhaustion” or “functional inhibition.” In contrast, PLR was able to reverse this “exhaustion” state, alleviate the excessive negative feedback, and restore the NF-κB pathway to its normal physiological dynamic balance. Poly ADP-ribose polymerase-1 (PARP1) plays a critical role in DNA repair. Upon DNA is damaged, PARP1 is activated and binds to the damaged site. Subsequently, PARP1 recruits multiple proteins involved in various aspects of DNA damage repair through poly(ADP)ribosylation (PARylation), thereby initiating the DNA repair mechanism (83). Prostaglandin-endoperoxide synthase 2 (PTGS2), also known as cyclooxygenase-2 (COX-2), catalyzes the conversion of arachidonic acid to prostaglandin precursors, primarily prostaglandin G2 (PGG2) and prostaglandin H2 (PGH2). These intermediates are subsequently metabolized to more stable, biologically active prostanoids, including prostaglandin E2 (PGE2) and prostacyclin (PGI2). In the kidney, COX2 is predominately expressed in renal medullary interstitial cells, cortical thick ascending limb cells, and macula densa-associated cells. Its products such as PGE2 and PGI2, play crucial roles in regulating glomerular filtration rate (GFR), renin release, and water/sodium excretion. Under pathological conditions, the expression of PGE2 and PGI2 are upregulated to maintain renal blood flow and GFR. However, sustained overexpression can promote renal inflammation and fibrosis (84, 85). Janus kinase 2 (JAK2) belongs to the family of non-receptor tyrosine kinases. When cytokines, primarily from the IL-6 family, bind to their receptors on the cell membrane, JAK2 is activated. This activation triggers the phosphorylation of the downstream molecule STAT3, thereby initiating the JAK2-STAT3 signaling pathway. This ubiquitous intracellular pathway plays critical roles in diverse biological processes, including cell proliferation, differentiation, apoptosis, and immune regulation. Additionally, this pathway is subject to negative regulation by several factors, such as suppressors of cytokine signaling (SOCS), protein inhibitors of activated STAT (PIAS), and protein tyrosine phosphatases (PTP), which control the magnitude and duration of signaling (86, 87). In this study, the expression of these five core target genes was upregulated in HUA rats, suggesting that renal tissues may have activated a negative feedback regulatory mechanism to maintain normal kidney function and prevent tissue damage. This observation was consistent with the HE staining results, which showed no obvious pathological changes in the renal tissues. PLR intervention effectively modulated gene expression, indicating that the negative feedback regulatory state in the kidneys may have been alleviated, allowing the expression of these genes to return to baseline physiological levels. This shift reflects the restoration of tissue homeostasis and may signify the initiation of repair processes. Nevertheless, definitive demonstration of this regulatory mechanisms will necessitate targeted in-vivo and in-vivo experiments.

## 5 Conclusion

**Figure 9:**
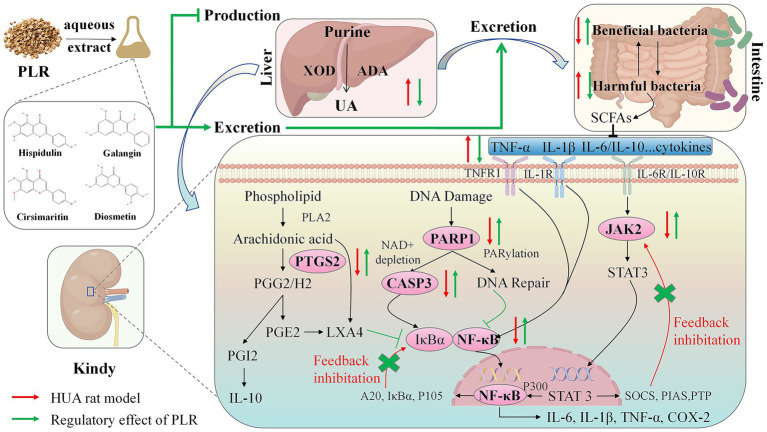
Schematic diagram illustrating the potential mechanism of PLR in hyperuricemic rats. In the liver, PLR reduces uric acid levels by inhibiting the activity of ADA and XOD. In the kidney and intestine, PLR exerts renoprotective effects and modulates the abundance of intestinal flora to promote the excretion of uric acid. Furthermore, in the kidney, PLR may upregulate the expression of CASP3, NF-κB, PTGS2, PARP1, and JAK2, alleviate compensatory negative feedback regulation and the restore tissue homeostasis.

In summary, this study investigated the preventive and therapeutic effects of PLR on HUA, with the primary mechanism illustrated in Figure 9. PLR could ameliorate serum uric acid concentrations by inhibiting the activity of uric acid synthase, and decreased inflammation and indicators of renal function, alleviate the hepatic and renal function in PO-induced hyperuricemia rats. Additionally, PLR altered the gut microbiota structure and increased the abundance of beneficial bacteria like Clostridium and Duncaniella. Integrated network pharmacology analysis coupled with PCR validation revealed that hispidulin, cirsimaritin, galangin, and diosmetin in PLR may modulate the expression of CASP3, NF-κB, PTGS2, PARP1, and JAK2. This modulation appears to alleviate compensatory negative feedback regulation in the kidneys and restore tissue homeostasis through the regulation of multiple signaling. Our findings offer valuable insights into the clinical application of the edible wild plant PLR in the management of HUA. Nevertheless, further investigations are required to fully elucidate two key aspects: (1) the isolation of these candidate compounds and their targeted *in vitro*/*in vivo* validation to definitively confirm their roles in anti-hyperuricemia; and (2) the underlying mechanisms, particularly the potential involvement of the intestinal flora in mediating PLR’s anti-hyperuricemic properties.

## Data Availability

The original contributions presented in the study are included in the article/Supplementary material, further inquiries can be directed to the corresponding authors.

## Footnotes

^1^ https://sciex.com.cn/

^2^ https://www.cnki.net/

^3^ https://pubmed.ncbi.nlm.nih.gov/

^4^ https://www.tcmsp-e.com/

^5^ https://www.bidd.group/TCMID/

^6^ http://bionet.ncpsb.org.cn/batman-tcm

^7^ https://pubchem.ncbi.nlm.nih.gov/

^8^ http://swisstargetprediction.ch/

^9^ https://www.genecards.org/

^10^ https://go.drugbank.com/

^11^ http://www.ncbi.nlm.nih.gov/omim

^12^ https://bioinfogp.cnb.csic.es/tools/venny/

^13^ https://cn.string-db.org/

^14^ https://davidbioinformatics.nih.gov/

^15^ https://www.bioinformatics.com.cn/

^16^ https://www.rcsb.org/
